# Differential methylation and expression patterns of microRNAs in relation to breast cancer subtypes among American women of African and European ancestry

**DOI:** 10.1371/journal.pone.0249229

**Published:** 2021-03-30

**Authors:** Zhihong Gong, Jianhong Chen, Jie Wang, Song Liu, Christine B. Ambrosone, Michael J. Higgins

**Affiliations:** 1 Department of Cancer Prevention and Control, Roswell Park Comprehensive Cancer Center, Buffalo, New York, United States of America; 2 Department of Biostatistics and Bioinformatics, Roswell Park Comprehensive Cancer Center, Buffalo, New York, United States of America; 3 Department of Molecular and Cellular Biology, Roswell Park Comprehensive Cancer Center, Buffalo, New York, United States of America; Cleveland Clinic Lerner Research Institute, UNITED STATES

## Abstract

Aggressive high-grade, estrogen receptor negative (ER-) breast cancer is more common among American women of African ancestry (AA) than those of European ancestry (EA). Epigenetic mechanisms, particularly DNA methylation and altered microRNA (miRNA) expression, may contribute to racial differences in breast cancer. However, few studies have specifically characterized genome-wide DNA methylation-based modifications at the miRNA level in relation to ER+ and ER- subtype, and their functional role in the regulation of miRNA expression, especially among high risk AA women. In this study, we evaluated DNA methylation patterns of miRNA encoding genes and their effect on expression in breast tumors from both AA and EA women. The genome-wide methylation screen identified a total of 7,191 unique CpGs mapped to 1,292 miRNA genes, corresponding to 2,035 unique mature miRNAs. We identified differentially methylated loci (DMLs: (|delta β|)>0.10, FDR<0.05) between ER- and ER+ tumor subtypes, including 290 DMLs shared in both races, 317 and 136 were specific to AA and EA women, respectively. Integrated analysis identified certain DMLs whose methylation levels were significantly correlated with the expression of relevant miRNAs, such as multiple CpGs within *miR-190b* and *miR-135b* highly negatively correlated with their expression. These results were then validated in the TCGA dataset. Target prediction and pathway analysis showed that these DNA methylation-dysregulated miRNAs are involved in multiple cancer-related pathways, including cell cycle G1-S growth factor regulation, cytoskeleton remodeling, angiogenesis, EMT, and ESR1-mediated signaling pathways. In summary, our results suggest that DNA methylation changes within miRNA genes are associated with altered miRNA expression, which may contribute to the network of subtype- and race-related tumor biological differences in breast cancer. These findings support the involvement of epigenetic regulation of miRNA expression and provide insights into the relations of clinical-relevant miRNAs to their target genes, which may serve as potential preventative and therapeutic targets.

## Introduction

Breast cancer is a heterogeneous disease, comprised of different clinical subtypes linked to disparate prognosis. However, as proposed by Anderson and colleagues [1], there are two primary etiologic subtypes, each associated with a distinct set of risk factors and genetic profiles that essentially correspond to estrogen receptor positive (ER+) and negative (ER-) disease, as an important marker for treatment options and prognosis. Compared to women diagnosed with ER+ breast cancer, those with ER- tumors in general have a poor prognosis, partly because of their aggressive phenotype and the lack of targeted therapy. High-grade, ER- breast cancer is more common among American women of African ancestry (AA) than those of European ancestry (EA). However, much less is known about the underlying causes of increased risk of ER- breast cancer in AA women.

Changes in DNA methylation have been recognized as one of the most common molecular alterations in cancer [2]. Aberrant methylation patterns have been frequently reported in breast cancer, with studies primarily focused on promoter regions of protein-coding genes (PCGs) [3–6]. Specific methylation patterns have been associated with high tumor grade and ER negativity of breast cancer [4, 7–9]. In our own studies and others, analyses restricted to CpG dinucleotides (CpGs) within and nearby PCGs, show differences in DNA methylation patterns in tumors by ER status and between AA and EA women [10–13]. These results suggest that aggressive breast cancer subtypes may be related to altered gene methylation, and that methylation patterns may differ by ancestry.

Although it is well known that more than 75% of the genome is actively transcribed, yielding a large number of non-coding RNAs (ncRNAs), the genome-wide methylation patterns of ncRNAs in breast cancer remain largely unknown. MicroRNAs (miRNAs) are a well-characterized class of small ncRNAs that are key regulators of PCG expression, through induction of mRNA degradation or interference with mRNA translation [14]. It is estimated that miRNAs regulate more than 50 percent of human genes and are found to play an essential role in the regulation of many biologic processes in the development of cancer, including cell differentiation, apoptosis, immunity, and proliferation [15]. Specifically in breast cancer, miRNA expression patterns in tumors have been linked to pathologic presentation and tumor subtypes [16–18], and specific miRNAs have been found to play essential roles in breast cancer invasion and metastasis [17, 19, 20]. Moreover, in a recent study, we observed differential miRNA expression patterns by ER status and between races [21].

As previous research has largely focused on the regulatory roles of miRNAs in cancer, their own regulation is not fully understood. Recent studies have shown that, similar to PCGs, miRNA encoding genes can be regulated by aberrant DNA methylation, and hence contribute to carcinogenesis [22, 23]. Given that a single miRNA can regulate expression of multiple target genes, alteration of methylation patterns of miRNAs may have a much broader effect than at other loci. A number of studies have reported altered methylation of miRNA genes in breast cancer, however, most of these studies have been limited by one or more key factors, such as focusing on only a small number of candidate miRNA genes, few studies on human breast tumor tissues, and lack of data on direct correlation between methylation and expression levels [24]. In addition, as we observed differences in miRNA expression patterns between ER- and ER+ tumors and between AA and EA women, there are no studies, however, that have specifically evaluated whether these differences are caused by alterations of DNA methylation patterns of CpGs within loci encoding miRNAs in breast cancer tissues from women of both races.

Therefore, in the current study, we specifically addressed aforementioned limitations of prior research. First, we characterized genome-wide DNA methylation patterns of CpGs within or near loci encoding miRNAs in breast tissues from both AA and EA women. Second, we examined DNA methylation alteration patterns at these loci in ER+ and ER- tumors from both races and compared the variability within and across race. Third, for a subgroup of tumors, for which we previously performed genome-wide miRNA expression profiling [21], we directly integrated DNA methylation and miRNA expression data to examine whether DNA methylation was associated with altered gene expression of corresponding miRNAs. Fourth, our results were then validated using The Cancer Genome Atlas (TCGA) dataset. Lastly, we performed target predication for the top CpG-miRNA pairs showing highly correlated methylation and miRNA expression confirmed in both our and TCGA dataset, and identified their enriched biological processes and signaling pathways.

Identification and quantification of genome-wide DNA methylation-based characteristics at the miRNA level in ER+ and ER- tumors may improve our understanding of the molecular basis for the phenotypic differences between these two primary subtypes of breast cancer. In addition, examining DNA methylation changes in aggressive ER- tumor commonly observed in AA women may provide insights into underlying causes of breast cancer racial disparities.

## Materials and methods

### Tissue samples and DNA methylation assay

As described previously, we conducted genome-wide DNA methylation profiling of fresh-frozen breast tumor samples from 58 AA and 80 EA women using the Illumina Infinium HumanMethylation450 Bead Chip platform, with subsequent analyses restricted to CpG loci within or near PCGs [10]. In the current analysis of these same samples, we focused on CpGs within loci encoding miRNAs. Briefly, breast tumor tissues were collected from patients who were treated at Roswell Park Comprehensive Cancer Center. Genomic DNA was isolated from banked specimens and stored at -80°C until use. The protocol was approved by the Roswell Park Institutional Review Board, with signed patient consent for use of remnant tissue for research. All samples were linked with detailed clinical information by the Roswell Park Biomedical Data Science Shared Resource.

Genome-wide DNA methylation analysis was carried out at Roswell Park Genetics Shared Resource using the Illumina Infinium HumanMethylation450 BeadChip platform, which interrogates > 485,000 CpG dinucleotides per sample at single-nucleotide resolution and covers 99% of RefSeq genes. In order to minimize the impact of batch effects, DNA samples from tumors were randomized on plates according to age, ancestry, and ER status. Following bisulfite treatment of DNA, subsequent steps for the methylation 450 BeadChip assay were carried out according to the manufacturer’s instructions.

### DNA methylation data processing and analysis

Hybridized and processed arrays were scanned using Illumina BeadArray Reader with High-Density (HD) Technology and BeadScan software. The raw intensities were then extracted using GenomeStudio, and the data summarized into BeadStudio IDAT files and processed by the minfi R package. The methylation level of each CpG site, expressed as a β value, ranges between (0, 1), with 0 for absent methylation and 1 for complete methylation. We applied rigorous sample and locus specific quality control criteria, SWAN normalization, and correction for batch effects [25]. We removed samples with poor detection p-values using the IMA package [26]. Probes that were ambiguously mapped and those shown to contain SNPs were also excluded from the analysis [27, 28], leaving the final dataset contained 276,108 CpG loci. We then identified all CpGs potentially associated with miRNA coding regions, as defined as any CpG loci located within 5kb of the miRNA coding region.

The unsupervised consensus clustering was performed on all unique miRNA-associated CpGs to examine overall methylation patterns in breast tumors, using the ConsensusClusterPlus R package (Bioconductor) with partitioning around medoids algorithm. We selected 100% item resampling (pItem), 90% probes resampling (pFeature), and a maximum evaluated (maxK) k of 6 so that cluster counts of 2,3,4,5,6 were evaluated. To identify clustering probe sets that have highly variable methylation patterns, 0.9 quantile of β value was used as a threshold, which yielded a set of 791 probes. The final consensus clustering was given by combining the ward.D linkage method and the Pearson correlation-based distance measurement. Differences in DNA methylation β-values for each probe were evaluated in tumor tissues by ER status within each race. The Wilcoxon rank-sum test was used to evaluate the statistical significance for each probe in each of the comparisons. To adjust for multiple comparisons, the false discovery rate (FDR) was computed using the Benjamini and Hochberg approach. Differentially methylated loci (DML) were defined as CpGs with an absolute mean β-value difference (|delta β|) at least 0.10 between subgroups and *FDR*-adjusted p < 0.05. All analyses were performed using R package.

### miRNA expression profiling and data processing

Among breast tumors included in the DNA methylation assay, miRNA-seq was performed on a subset of tumors, including 58 tumors (29 AA and 29 EA women). As described previously [21], 1 μg RNA was used for small RNA cDNA library preparation and then samples were sequenced on the Illumina HiSeq 2500 platform using high output 50-cycle single read sequencing. The 3′ adapter in the reads was trimmed by cutadapt (version 1.12). The trimmed reads were aligned to human reference genome (hg19) by Burrows-Wheeler Aligner (version 0.7.15) with no mismatches allowed. The miRNA profiling pipeline developed by the British Columbia Genome Sciences Centre was implemented to quantify miRNA expression. The miRNA quantification data were generated including expression levels for the known mature miRNAs for all samples.

### The Cancer Genome Atlas (TCGA) data processing and analysis

To validate our findings, TCGA DNA methylation and miRNA expression data were downloaded from GDAC Broad Institute (http://gdac.broadinstitute.org/runs/stddata 2016_01_28/data/BRCA/20160128/). Data generated using the same platform as the current study were included in this analysis. Patient and breast cancer clinical features were retrieved, such as age, race, and ER status. All data were processed and analyzed following the same pipeline as used in the current study (above).

### DNA methylation status and association with miRNA expression

To examine whether DNA methylation status is associated with expression of corresponding miRNAs, we paired each CpG site with the corresponding miRNA, defined as probes located within 5Kb of a miRNA gene, as one CpG-miR Pair. Unique CpG-miR pairs were identified, in which one miRNA can be paired with multiple CpG sites, and similarly one CpG site can be paired with multiple miRNAs. Spearman’s nonparametric correlation analysis was used to compute the correlation coefficient (rho) between methylation levels (beta values) and miRNA expression (log counts per million, logCPM) for each pair. Correlations were considered significant as *P*<0.05. Correlation analyses were carried out separately for tumors from AA and in EA women.

### miRNA target gene prediction and pathway analysis

For several top candidate CpG methylation-correlated-miRNAs identified in our study and validated in TCGA dataset, we further performed target prediction and pathway analysis using MetaCore (Clarivate Analytics), an integrated knowledge-based platform for comprehensive functional analysis of OMICs data and gene list. This method is based on a high-quality, manually curated database of molecular interactions, molecular pathways, and gene-disease associations. Specifically, in this analysis, we identified target genes of these miRNAs and their enriched biological processes and pathways within gene sets. MetaCore employs a hypergeometric model to determine significance of functional relationships. Functions with *FDR*-adjusted p<0.05 were considered significant.

## Results

### DNA methylation alterations at miRNA-associated loci in breast tumor tissue from AA and EA women

**Fig 1:**
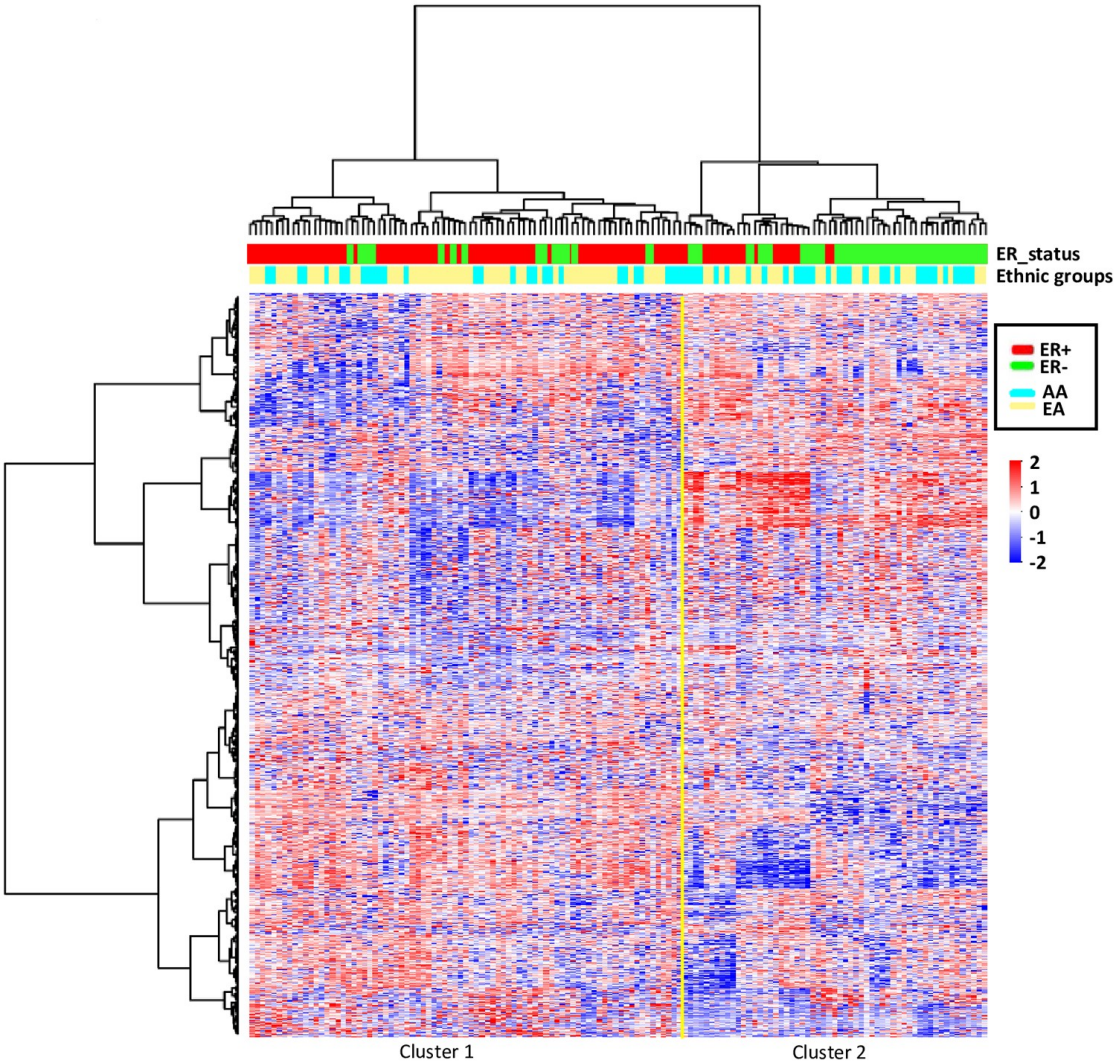
Unsupervised consensus cluster analysis of miRNA methylation patterns of breast tissues. Two major clusters were identified. Cluster 1 contains primarily ER+ breast tumors; cluster 2 is enriched for ER- tumors.

Based on the criterion that for a CpG locus to be associated with a miRNA gene, it must be located within 5kb of a miRNA coding locus, a total of 7,191 unique CpGs were identified. These CpGs were mapped to 1,292 miRNA genes, which correspond to 2,035 unique mature miRNAs based in the latest release of miRBase version 22 (http://www.mirbase.org/). As shown in Fig 1, unsupervised consensus clustering analysis of the methylation levels at these miRNA-associated CpG loci identified two major clusters. Cluster 1 contains primarily ER+ breast tumors while cluster 2 is enriched for ER- tumors. Age and tumor characteristics of breast cancer cases in AA and EA women are presented in Table 1.

**Table 1:** Characteristics of patients with breast cancer in current study.

Factors	AA (n = 58, %)	EA (n = 80, %)
Age		
<50	18 (32)	22 (28)
50–68	20 (34)	29 (36)
>68	20 (34)	29 (36)
Estrogen Receptor Status		
Negative	26 (45)	25 (31)
Positive	32 (55)	55 (69)
Progesterone Receptor Status		
Negative	31 (53)	41 (51)
Positive	27 (47)	39 (49)
HER2 Status^1^		
Negative	47 (87)	44 (59)
Weak	1 (<1)	4 (3)
Strong	7 (13)	26 (48)
Histological Grade^2^		
I (well differentiated)	0	2 (3)
II (moderately differentiated)	13 (23)	8 (10)
III (poorly differentiated)	44 (77)	68 (87)

^1^ HER-2 status missing for 3 AA and 6 EA women.

^2^ Histologic grade data missing for 1 AA and 2 EA women.

**Fig 2:**
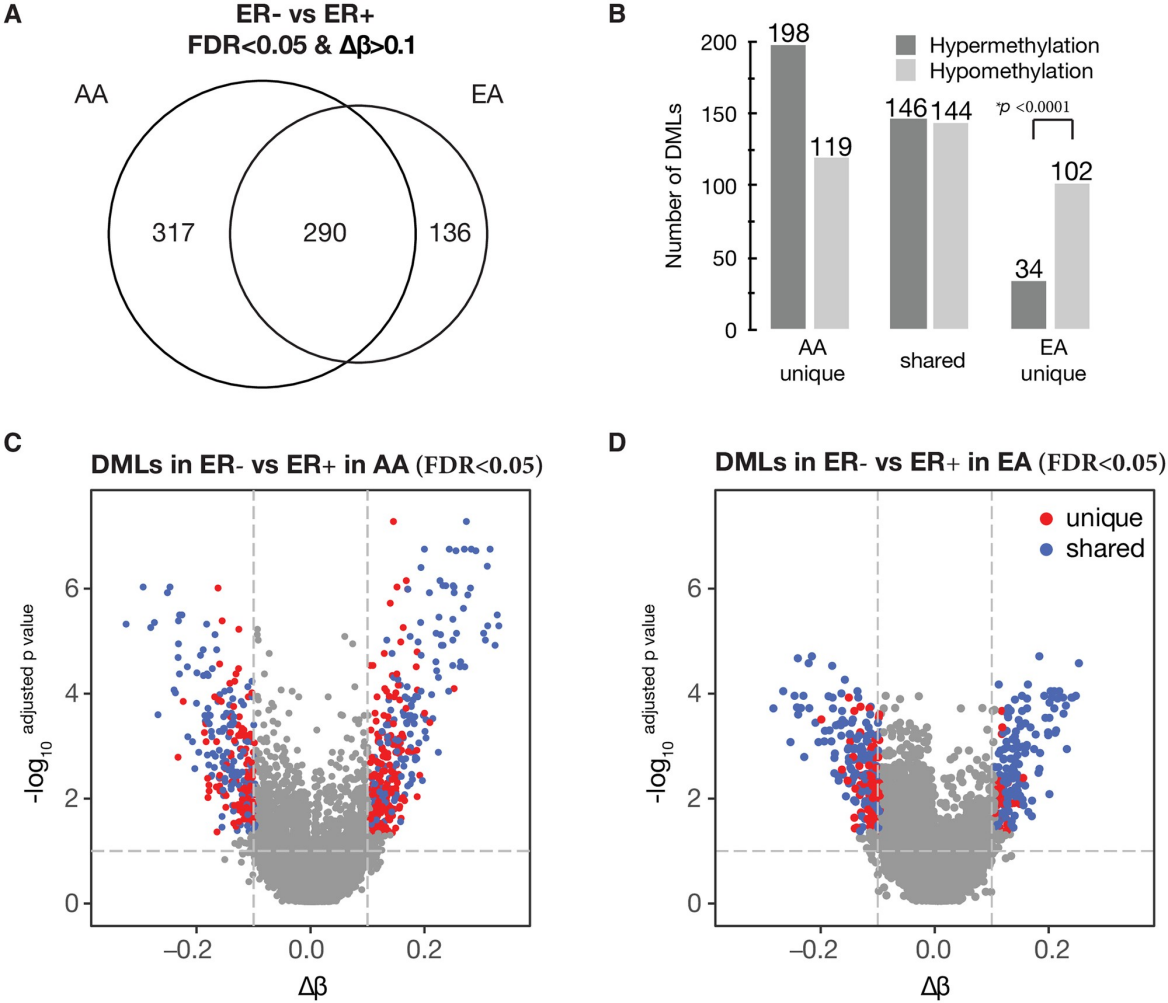
The differentially methylated loci (DML: |delta β|≥0.10; *FDR*<0.05) between ER- and ER+ tumors in AA and EA women. (A) Venn diagram showing the number of DMLs between ER- vs. ER+ tumor specific to EA women (n = 136), common in both AA and EA women (n = 290), and specific to AA women (n = 317). (B) The histogram shows the differences in proportions (*P*<0.05) of hyper- and hypo- methylated by ER-DMLs in AA-specific, AA-EA Shared, and EA-specific groups. (C and D) Volcano plots showing distributions of hyper- and hypo- methylated DMLs between ER- vs. ER+ tumor in AA (C) and in EA women (D). Dots plotted in red represent DMLs specific to either AA or EA women; Dots plotted in blue represent DMLs shared in both AA and EA women; red or blue dots in the right panel represent for hypermethylated and those in the left panel for hypomethylated DMLs. Dots plotted in grey are not DMLs in either AA or EA women. The mean methylation difference (|delta β|) in ER- versus ER+ tumors is on the X axis, and the Y axis shows the log 10 of FDR (adjusted *P*-value).

As no known studies have specifically examined genome-wide DNA methylation patterns of miRNA encoding genes in breast tumors from AA populations, and there is a clear pattern of ER-subtype differences (Fig 1), in the current study, we focused on analyses to identify differentially methylated loci (DML**:** |delta β|≥0.10; *FDR*<0.05) between ER- and ER+ tumors in AA and EA women, separately. As shown in Fig 2A and 2B, we identified 607 DMLs in tumors from AA women, while there were 426 DMLs in tumors from EA women. Among DMLs identified in ER- versus ER+ tumors from either AA or EA women, 290 DMLs (146 hyper- and 144 hypo- methylated) were identified in both races, 317 were specific to AAs (198 hyper- and 119 hypo- methylated), and 136 were specific to EAs (34 hyper- and 102 hypo-methylated). We observed significant differences in proportions of hyper- versus hypo-methylated race-specific DMLs (*P*<0.0001). We further presented these DMLs in the volcano plot for AA women (Fig 2C) and for EA women (Fig 2D), which clearly demonstrated a significantly higher frequency of hypermethylated loci in tumors from AA women compared to EA women. The full list of these DMLs by ER subtype for each group is shown in S1 Table.

### Validation of DMLs in the TCGA dataset

We validated DNA methylation for DMLs identified above using data from TCGA, which included 141 AA and 396 EA cases in this analysis (S2 Table). Data on 537 (out of 607 DMLs in AAs) and 385 (out of 426 DMLs in EAs) CpGs were available in the TCGA dataset. As shown in S1 Fig, we observed a high concordance between DMLs detected in our data and those identified using the TCGA dataset, i.e., all probes showed the same direction of methylation change by ER status, although in TCGA dataset, methylation changes of 16 DMLs in AAs and the 3 DMLs in EAs did not reach the statistical significance (dots in red).

### Impact of aberrant DNA methylation on miRNA expression

**Fig 3:**
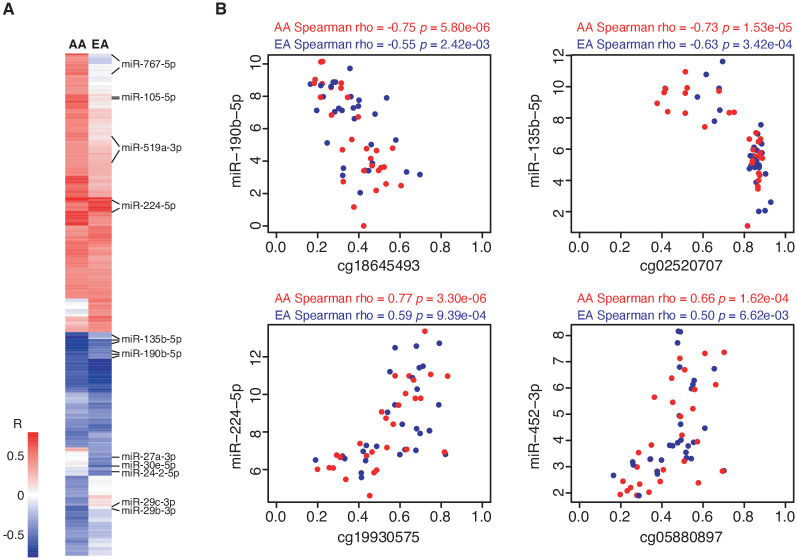
Correlation coefficients between methylation and miRNA expression levels on by ER-DMLs and their corresponding miRNAs. (A) The heatmap shows the Spearman’s correlation coefficients (rho) between each CpG-miR pair (*P*<0.05) in AA and EA tumors. Red bars represent positive correlation and blue bars represent negative correlation. (B) The Scatter plots relate the methylation and expression levels on selected top correlated CpG-miR pairs including specific probes with miR-190b-5p, miR-135b-5p, miR-224-5p, and miR-452-3p. For each CpG-miR pair, the methylation level (beta value) is on the X axis, and the expression level (log counts per million, logCPM) of corresponding miRNA is on Y axis. The Spearman’s correlation coefficient (rho) of each CpG-miR pair for AA and EA tumors was presented in the figure.

To examine whether these DMLs are associated with expression of their corresponding miRNAs, we paired each CpG with the corresponding miRNA defined as within 5Kb window, with a total of 1,408 unique CpG-miR pairs identified in our data. In cancers from AA women, we identified 224 (94 negative and 130 positive) significant correlations (*P*<0.05) between methylation and expression levels, involving 136 unique CpGs at which differential methylation was associated with expression of 124 mature miRNAs from 97 unique miRNA genes. In tumors from EA women, we found 97 (45 negative and 52 positive) correlations, corresponding to 70 CpGs and 64 mature miRNAs from 51 unique miRNA genes. The correlation coefficients for each of these significant CpG-miR pairs are shown in Fig 3A, with detailed information on the CpG loci, mature miRNAs, and their correlation coefficients listed in S3 Table. Notably, in both race groups, significant negative correlations for multiple CpG loci were observed for *miR-190b* and *miR-135b*, whereas positive correlations were observed for members of the *miR-224/miR-452* cluster. As shown in Fig 3B, scatter plots showed statistically significant correlation between the methylation levels of several CpG loci in the *miR-190b*, *miR-135b*, and *miR-452/-224* with the corresponding miRNA expression. We also identified CpGs correlated with the expression of corresponding miRNAs in either AA or EA tumors, such as members of the *miR-105/miR-767* cluster, which were specific to AA tumors, while *miR-27a*, *-30e*, *-29c/-29b* were specific to EA tumors. These results suggest that DNA methylation changes appeared to drive miRNA expression alterations, that these correlations were somewhat stronger in AAs than in EAs, and that some correlations are specific to AA or EA tumors.

We further validated these findings in the TCGA dataset. The majority of correlations identified in our study showed consistent directions in TCGA, with detailed information listed in S4 Table, and scatter plots showing significant correlations for multiple CpGs with their corresponding miRNAs, i.e., the *miR-190b*, *miR-135b*, *miR-224/-452* (S2 Fig). As shown, the methylation level of a CpG mapped within the *miR-190b* locus was negatively correlated with expression levels of the mature miR-190b-5p, in which high methylation levels were associated with low expression levels in both our and TCGA data. Similar findings were observed for multiple CpGs of *miR-135b*. These results further confirmed that DNA methylation modifications in miRNA encoding genes play regulatory role in their expression.

### miRNA target genes and pathway analysis

**Fig 4:**
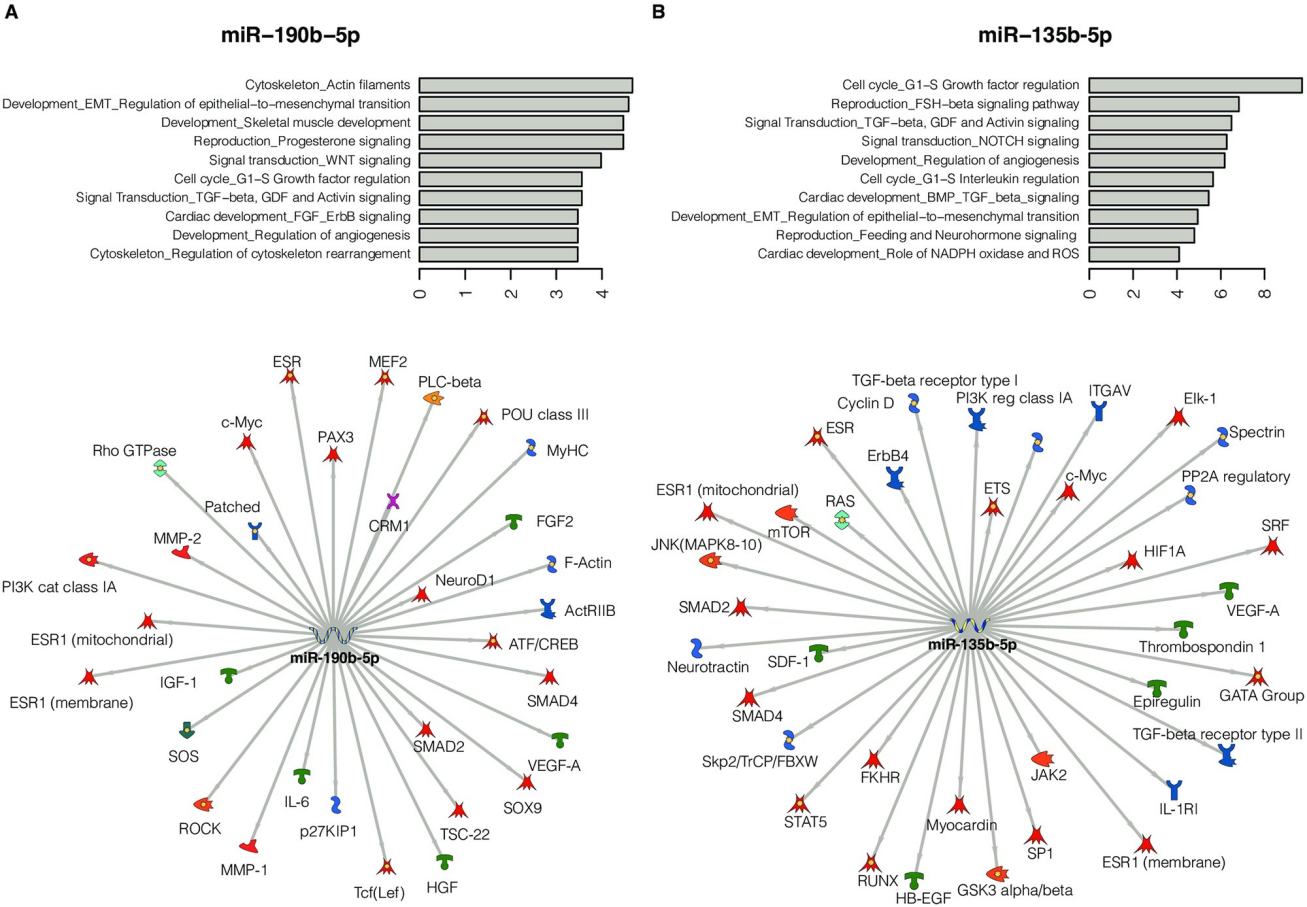
Functional enrichment analysis on targets from miR-135b-5p and miR-190b-5p using MetaCore. (A) Target gene network and top ten pathway networks for miR-190b-5p. (B) Target gene network and top ten pathway networks for miR-135b-5p.

To investigate the biological relevance of these findings, we performed target prediction and pathway enrichment analysis using MetaCore, focusing on four top candidate miRNAs at which DNA methylation was highly correlated with miRNA expression in both our and TCGA dataset, including miR-135b-5p, miR-190b-5p, and miR-224-5p/miR-452-3p. Details on molecular process networks, and targets for each miRNA are presented in S5 Table. These miRNAs are involved in multiple cancer-related pathways, indicating their potential role in various key molecular processes related to and beyond ER biology. As shown in Fig 4, top enriched pathway and process networks related to miR-190b-5p and miR-135b-5p include cell cycle G1-S growth factor or interleukin regulations, cytoskeleton remodeling, angiogenesis, EMT, and others such as signal transduction TGF-β, Wnt, NOTCH, and ESR1-mediated signaling pathways. Targets from these process networks were identified, including various transcription factors and protein kinase, such as *ESR1*, *GSK3β*, *MMP-2*, *MMP-9*, *TGF-β*, *mTOR*, *and PI3KCA*.

## Discussion

DNA methylation at miRNA genes has been reported as a mechanism that may cause dysregulation of miRNA expression and consequently associated with breast cancer risk and progression. However, few studies have specifically examined this epigenetic modification at miRNA genes in relation to ER+ and ER- breast cancer, and its functional role in the regulation of miRNA expression, especially in AA women who are at high risk of aggressive, ER- disease. In this study, we evaluated methylation patterns of miRNA genes and their effect on miRNA expression in breast tumors from both AA and EA women. The genome-wide methylation screen identified a set of DNA methylation loci within 5Kb of miRNA genes that were differentially methylated between ER- and ER+ tumor subtypes in tumors from both races, or specific to AA or EA women. Integrated analysis of DNA methylation and miRNA expression identified certain DMLs whose methylation levels were significantly correlated with the expression of relevant miRNAs. Thus, DNA methylation changes associated with altered miRNA expression may contribute to the network of molecular differences of ER subtypes of breast cancer and may differ by race.

We identified many DMLs that can significantly differentiate ER- and ER+ tumor subtypes. Some of the top DMLs mapped to, or were in the vicinity of, miRNAs that are differentially expressed in ER- versus ER+ tumors, such as multiple CpGs in *miR-135b*, *-190b*, and *-224*, as shown in our previous study and in other studies [21, 29]. Many other DMLs were novel relative to ER subtypes, such as CpGs associated with *miR-125b1*, *-2053*, *-3132*, and *-4736*. Some of these DMLs were in common between races and others were specific to AA or EA women. We also noted that there were more DMLs by ER subtypes in AA tumors compared to EA tumors. Moreover, while ER-subtype DMLs common to both races showed similar proportions of hyper- and hypo-methylated loci, interestingly, we noted significant differences in proportions for race-specific DMLs. We observed a higher frequency of hypermethylated miRNA genes in ER- compared to ER+ tumors from AA women, but a lower frequency of hypermethylation in ER- versus ER+ tumors from EA women. Consistent with our findings for tumors from EA women, several studies focused on DNA methylation of coding genes observed a higher frequency of hypermethylated genes in ER+ tumors [8, 30, 31]. One explanation for this may be that ER-related signaling molecules may modify the activity of DNA methylation transferases that catalyze the transfer of a methyl group to a DNA segment and/or enzymes involved in demethylation of DNA (e.g., TET1-3, APOBEC), thus lead to the specific gain of DNA methylation in ER+ tumors. By contrast, in tumors from AA women, we observed that there were more DMLs associated with ER tumor subtypes and that there was a higher frequency of hypermethylated miRNA loci in ER- tumors, which warrant further investigations. Nevertheless, the majority of these DMLs were confirmed in TCGA data, suggesting a differential DNA methylation pattern of miRNA encoding genes relevant to breast cancer subtypes and race. Although we identified differentially methylated loci of miRNA genes by tumor subtypes and between races, we are unable to control non-biological factors, which may contribute to the differences in DNA methylation [32]. Therefore, further validation of these findings and more in-depth research into the possible causes is important.

Another goal of our work was to identify DNA methylation-associated changes in miRNA expression that may be involved in phenotypic differences in breast cancer subtypes in both AA and EA women. Leveraging the availability of both genome-wide DNA methylation and miRNA expression data from the same patients, we were able to directly link both types of data and examined whether altered DNA methylation at specific CpG loci was associated with the expression of neighboring miRNAs. As expected, we observed that altered methylation at certain DMLs was negatively correlated with expression of corresponding miRNAs in breast tumors of both AA and EA women. Several top correlated CpG-miR pairs include multiple loci within the *miR-190b* and *miR-135b*. *MiR-190b* has been reported to be highly upregulated in ER+ compared to ER- breast cancers from several other studies including our own [21, 33–35]. In the present study, our results provide evidence that differences in *miR-190b* expression in breast cancer subtypes are associated with methylation alterations. This observation was consistent with results from our analysis of TCGA dataset and from a recently published study [35]. Results from studies also suggest that expression of *miR-190b* in primary tumors may play a role with breast cancer recurrence, tamoxifen resistance, breast cancer and overall survival, but its exact role in carcinogenesis remains to elucidate [33, 34, 36]. Interestingly, as low expression is associated with poor survival, we observed the lowest *miR-190b* expression in ER- tumors from AA women among all tumor-race subgroups, which may contribute to the poor survival observed in this population. By contrast, we observed CpG promoter hypomethylation and upregulation of *miR-135b* in ER- versus ER+ tumors. Consistent with these results, several studies have reported *miR-135b* was upregulated in ER- or triple negative breast cancers and its overexpression promotes breast cancer progression and metastasis through regulation of *ERα*, *AR*, and other genes such as *LATS2*, *TGFβ*, *WNT*, and *HIF1AN* [37–41]. Taken together, confirmed in TCGA data, these consistent negative correlations between CpG methylation and miRNA expression support the notion that, at least in some cases, altered miRNA expression may occur through mechanisms involving loss or gain of promoter DNA methylation during early stages of carcinogenesis and contribute to the development of specific subtypes of the disease. These DNA methylation-dysregulated network of miRNA genes could be clinically relevant predictive markers and served as potential new therapeutic targets of anticancer therapy. It may be possible to restore the functionality of the dysregulated miRNA network through epigenetic drugs. Future study is necessary to fully understand the mechanisms of underlying biological functions and their clinical implications.

Although most studies have primarily focused on the relationship of DNA methylation and negative control of gene expression, we revealed positive correlations between methylation and expression for many CpG-miRNA pairs, similar to what has been consistently reported in cancer studies that investigate correlation patterns between CpG methylation and protein-coding gene expression at the genome-wide scale [42]. Notably, we found that DNA methylation of multiple loci within the *miR-224/miR-452* cluster were positively associated with miRNA expression in both races. These two intronic miRNAs are located within a locus on chromosome Xq28 encompassing *GABRE* gene. We observed hypermethylation at these CpG loci and increased *miR-224/miR-452* expression in ER- compared to ER+ tumors. Studies have reported both up- and down-regulation of *miR-224* in various cancers, suggesting its cell type-specific expression and function [43–45]. Specifically, in breast cancer, and consistent with our study, one study reported increased *miR-224* expression in both triple negative breast cancer cell lines and tumor tissues, and that its knockdown in aggressive triple negative breast cancer cells reduced proliferation, migration and invasion [46]. In addition, we observed significant positive correlations between methylation of multiple CpGs and expression of *miR-767* in AA women, which warrants further investigation. These results suggest the existence of more diverse mechanisms of epigenetic regulation, which may involve DNA methylation of insulator or repressor elements, transcription factor binding, chromatin and histone modifications during carcinogenesis. Such complexity warrants further investigation of these miRNAs and findings could provide implications for understanding the molecular basis for the phenotypic differences by breast cancer subtype and race.

As we mentioned above, we found that altered DNA methylation and expression of a number of miRNAs were associated with specific tumor subtypes (ER- vs. ER+) and differed by race. To investigate their biological functions relevant to tumor aggressiveness and progression, we further performed target prediction and enrichment analysis for several highly, significant methylation-dysregulated miRNAs, including *miR-190b* and *miR-135b*. Several important metastasis- and cancer-related pathways were enriched, including cell cycle regulation, angiogenesis, EMT, and Wnt, TGF-β and ESR1-mediated signaling. Specifically, we found that these miRNAs potentially participate in these pathways through interactions with mRNA targets transcribed from genes such as *ESR1*, *GSK3β*, *MMP-2/-9*, *TGFβ*, *mTOR*, *SMAD4*, *c-Myc*, *HIF1α*, and *PI3KCA*, some of which have been shown to regulate various signaling pathways and cellular functions in breast carcinogenesis [47–50]. These results suggest that these miRNAs target many key genes and work as a module, contributing to breast cancer phenotypic differences and aggressiveness. We speculate that differences in these miRNA-target gene networks may play a role for the high risk of developing aggressive breast cancer subtypes observed in AA women. Further experimental validation will be needed to confirm these findings.

## Conclusions

In summary, based on genome-wide profiling of tumor DNA methylation and microRNA expression, our results suggest that DNA methylation patterns in miRNA encoding genes differ between breast cancers according to cancer subtype and race, and that this altered methylation may affect miRNA expression. Further pathway analysis identified their potential role in modulating cancer-related key biological processes. These findings provide the basis for further functional analyses in experimental studies, which are likely to further inform the underpinning of subtype- and race-related tumor biological differences in breast cancer. Our study sheds light on the epigenetic regulation of miRNA expression and provide insights into the relations of clinical-relevant miRNAs to their target genes, which may serve as potential preventative and therapeutic targets.

## Supporting information

S1 Table Tumor characteristics among patients in TCGA cohort. (DOCX)

S2 Table The list of AA-specific, AA- and EA-shared, EA-specific DMLs by ER status. (XLSX)

S3 Table Correlations on significant CpG-miR pairs in the current study. (XLSX)

S4 Table Correlation validation in TCGA cohort. (XLSX)

S5 Table Enrichment analysis on targets from the selected top methylation-associated miRNAs. (XLSX)

S1 Fig Validation of DMLs identified in the current (Gong) study using TCGA cohort. (A and B) Scatter plot of delta beta value of available DMLs from Gong (x-axis) versus TCGA (y-axis) in AAs (A) and in EAs (B). DMLs identified in both data with consistent direction of methylation changes and FDR-adjusted *P*<0.05 are plotted as black dots. DMLs identified in the Gong study with consistent direction of methylation changes but did not reach statistical significance in TCGA data (dots in red).
(PDF)

S2 Fig Correlation validation of selected top CpG-miR pairs in TCGA cohort. The Scatter plots relate the methylation and expression levels on selected top correlated CpG-miR pairs including specific CpGs with miR-190b-5p, miR-135b-5p, miR-224-5p, and miR-452-3p. For each CpG-miR pair, the methylation level (beta value) is on the X axis, and the expression level (log counts per million, logCPM) of corresponding miRNA is on Y axis. The Spearman’s correlation coefficient (rho) of each CpG-miR pair for AA and EA tumors was presented in S2 Fig.
(PDF)
