# Supplementary material for: Differential methylation and expression patterns of microRNAs in relation to breast cancer subtypes among American women of African and European ancestry
Source: PLoS One. 2021 Mar 30;16(3):e0249229. doi: 10.1371/journal.pone.0249229 (PMC8009363; doi:10.1371/journal.pone.0249229)
Supplement: S1 Table — (DOCX) [file pone.0249229.s001.docx]

| S1 Table. Tumor characteristics among patients included in TCGA cohort | | |
| --- | --- | --- |
| Factors | AA (n=141, %) | EA (n=396, %) |
| Age |  |  |
| <50 | 44 (31) | 99 (25) |
| 50-68 | 60 (43) | 187 (47) |
| >68 | 36 (26) | 109 (28) |
| Estrogen Receptor Status |  |  |
| Negative | 56 (40) | 64 (16) |
| Positive | 85 (60) | 332 (84) |
| Progesterone Receptor Status |  |  |
| Negative | 74 (52) | 93 (24) |
| Positive | 67 (48) | 300 (76) |
| HER2 Status |  |  |
| Negative | 28 (20) | 100 (25) |
| Strong | 31(22) | 71 (18) |
| Weak | 11 (8) | 26 (7) |
| Unknown | 71 (50) | 198 (50) |
| Histological Grade |  |  |
| I (well differentiated) | 24 (17) | 70 (18) |
| II (moderately differentiated) | 88 (62) | 210 (53) |
| III/IV (poorly differentiated) | 29 (21) | 114 (29) |
| *Numbers may not add up to the total number of participants because of missing values. | | |
