# Supplementary figures and images for: Differential methylation and expression patterns of microRNAs in relation to breast cancer subtypes among American women of African and European ancestry
Source: PLoS One. 2021 Mar 30;16(3):e0249229. doi: 10.1371/journal.pone.0249229 (PMC8009363; doi:10.1371/journal.pone.0249229)

**A**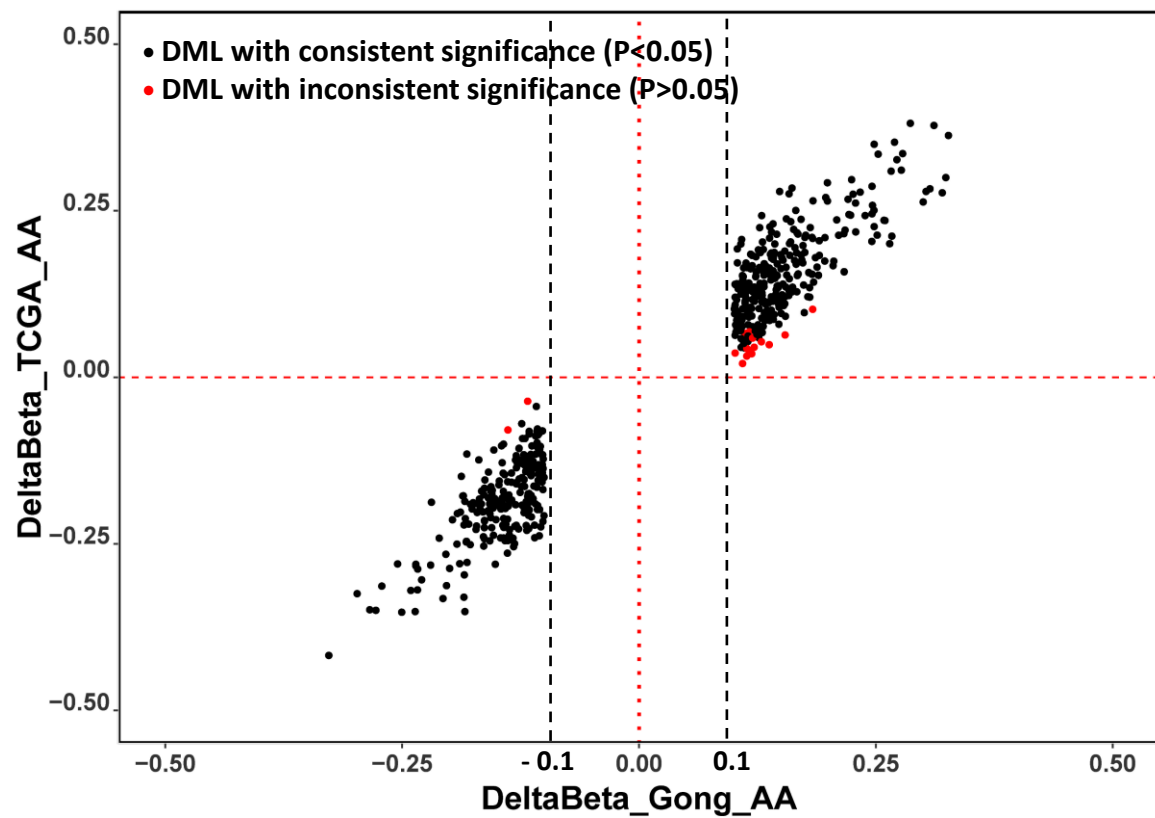**B**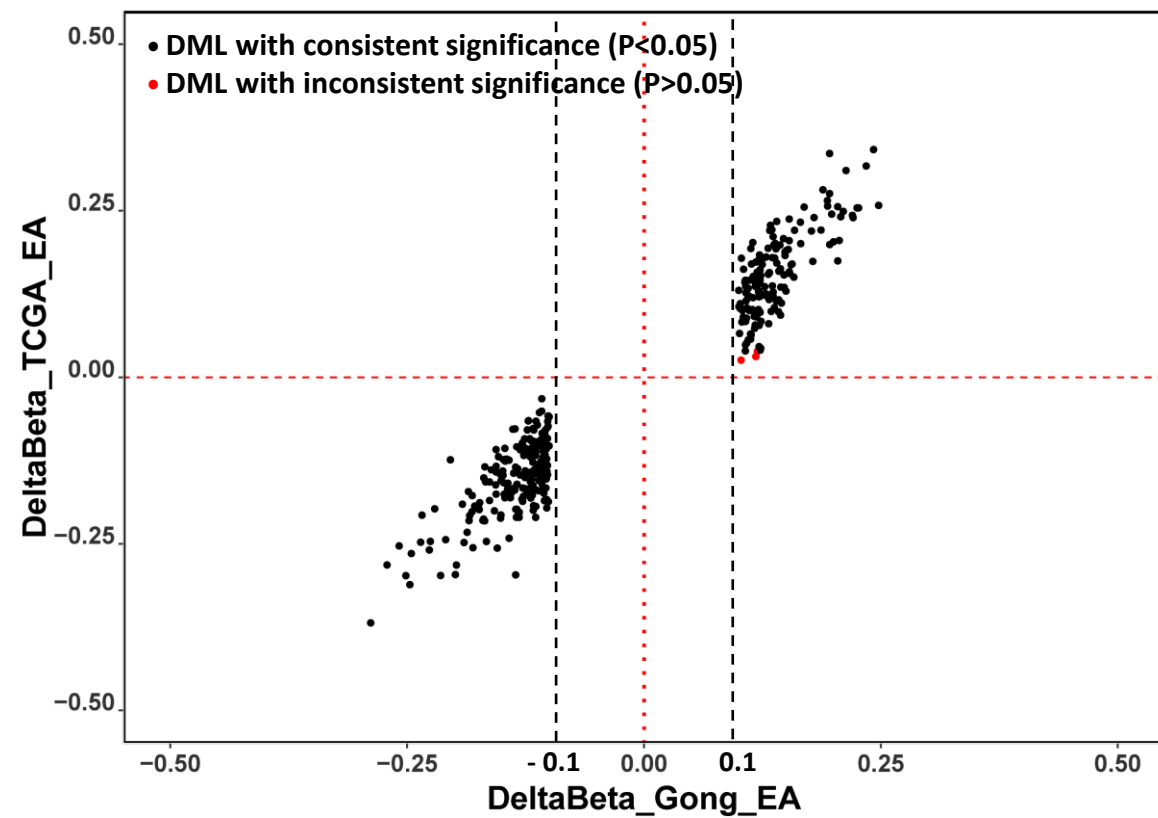

Supplement: S1 Fig — (A and B) Scatter plot of delta beta value of available DMLs from Gong (x-axis) versus TCGA (y-axis) in AAs (A) and in EAs (B). DMLs identified in both data with consistent direction of methylation changes and FDR-adjusted P<0.05 are plotted as black dots. DMLs identified in the Gong study with consistent direction of methylation changes but did not reach statistical significance in TCGA data (dots in red). (PDF) [file pone.0249229.s006.pdf]

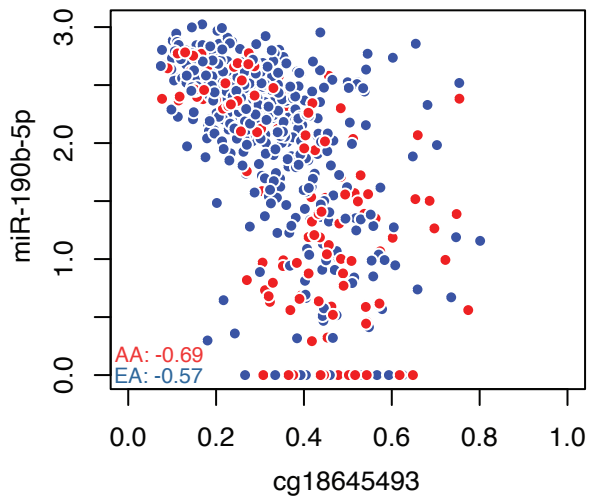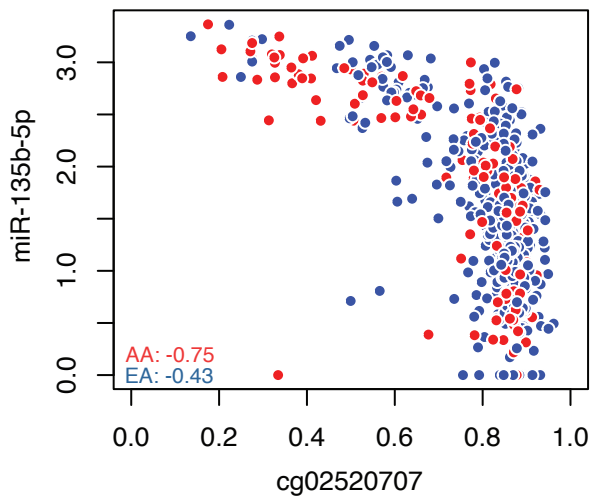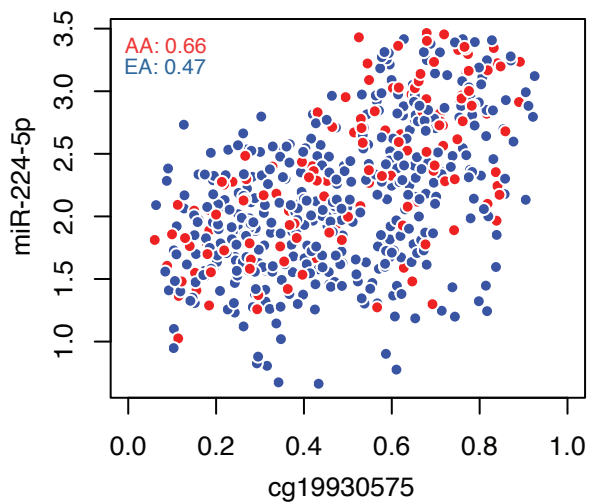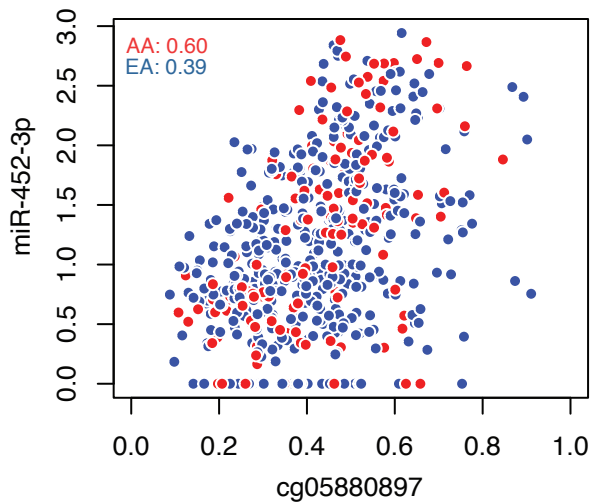

Supplement: S2 Fig — The Scatter plots relate the methylation and expression levels on selected top correlated CpG-miR pairs including specific CpGs with miR-190b-5p, miR-135b-5p, miR-224-5p, and miR-452-3p. For each CpG-miR pair, the methylation level (beta value) is on the X axis, and the expression level (log counts per million, logCPM) of corresponding miRNA is on Y axis. The Spearman’s correlation coefficient (rho) of each CpG-miR pair for AA and EA tumors was presented in S2 Fig. (PDF) [file pone.0249229.s007.pdf]
